# Administration of AAV-Alpha Synuclein NAC Antibody Improves Locomotor Behavior in Rats Overexpressing Alpha Synuclein

**DOI:** 10.3390/genes12060948

**Published:** 2021-06-21

**Authors:** Yun-Hsiang Chen, Kuo-Jen Wu, Wei Hsieh, Brandon K. Harvey, Barry J. Hoffer, Yun Wang, Seong-Jin Yu

**Affiliations:** 1 Department of Life Science, Fu-Jen Catholic University, New Taipei City 24205, Taiwan; 125648@mail.fju.edu.tw; 2 Center for Neuropsychiatric Research, National Health Research Institutes, Zhunan 35053, Taiwan; kjwu@nhri.edu.tw (K.-J.W.); hsiehwei@nhri.edu.tw (W.H.); ywang@nhri.edu.tw (Y.W.); 3 National Institute on Drug Abuse IRP, NIH, Baltimore, MD 21224, USA; BHarvey@intra.nida.nih.gov; 4 Department of Neurosurgery, School of Medicine, Case Western Reserve University, Cleveland, OH 44106, USA; bjh82@case.edu

**Keywords:** AAV, synuclein, Parkinson’s disease, immunotherapy

## Abstract

Accumulation of α-Synuclein (αSyn) in nigral dopaminergic neurons is commonly seen in patients with Parkinson′s disease (PD). We recently reported that transduction of intracellular single-chain intrabody targeting the 53–87 amino acid residues of human αSyn by recombinant adeno associated viral vector (AAV-NAC32) downregulated αSyn protein in SH-SY5Y cells and rat brain. This study characterizes the behavioral phenotype and dopaminergic protection in animals receiving AAV-NAC32. Our results show that adult DAT-Cre rats selectively overexpress αSyn in nigra dopaminergic neurons after local administration of AAV-DIO-αSyn. These animals develop PD-like phenotype, including bradykinesia and loss of tyrosine hydroxylase (TH) immunoreactivity in substantia nigra pars compacta dorsal tier (SNcd). An injection of AAV-NAC32 to nigra produces a selective antibody against αSyn and normalizes the behavior. AAV-NAC32 significantly increases TH, while reduces αSyn immunoreactivity in SNcd. Altogether, our data suggest that an AAV-mediated gene transfer of NAC32 antibody effectively antagonizes αSyn-mediated dopaminergic degeneration in nigra, which may be a promising therapeutic candidate for synucleinopathy or PD.

**Highlights**

- Overexpression of αSyn in nigra DA neurons induces bradykinesia and neurodegeneration in rats.
- Injection of AAV-NAC32 produces a selective antibody against αSyn in nigra.
- AAV-NAC32 normalizes the behavior and improves the survival of nigra dopaminergic cells.
- AAV-NAC32 effectively antagonizes αSyn-mediated dopaminergic degeneration in nigra.

## 1. Introduction

Parkinson’s disease (PD) is the second most common neurodegenerative disease, and is characterized by the loss of dopaminergic (DA) neurons in the substantia nigra pars compacta. The major histopathology in PD is the formation of fibrillar aggregate or Lewy bodies in nigra. α-Synuclein (αSyn), a 140 amino acid protein, is the primary component in the Lewy body and has been reported genetically linked to familial PD [1]. Overexpressing wildtype αSyn resulted in dopaminergic neurodegeneration and motor deficits in transgenic mice [2].

αSyn protein is composed of three distinct regions: (1) A highly conserved amino-terminal domain (residues 1–60), which forms amphipathic α-helical structures on binding to cellular membranes, (2) a central hydrophobic region (61–95 residues) termed the nonamyloidal component (NAC), which regulates the axonal transport of αSyn [3] and is essential for αSyn aggregation [4], and (3) a highly negatively charged C-terminus (96–140), in which most of the post-translational modifications are involved [5]. Two other proteins in the same synuclein family are βSyn (134 amino acids) and γSyn (127 amino acids). βSyn and γSyn share high sequence homology with αSyn. These synucleins, however, are not found in the Lewy body and are less involved in the pathology of PD. The major structural difference between αSyn and βSyn is in the NAC region. βSyn is missing an 11-residue stretch (73–84) in the NAC and is more resistant to aggregation [6]. 

Several studies have examined the effectiveness of antibody-based immunotherapy in synuclein-mediated neurodegeneration (or synucleinopathy). Application of specific scFv (or single-chain variable fragment) antibody against fibrillar αSyn attenuated αSyn-mediated aggregation and toxicity in SH-SY5Y cells [7]. Systemic administration of monoclonal antibody (9E4) against the C-terminus of αSyn (10 mg/kg, i.p., weekly × 6 months) improved motor and water maze learning behavior and promoted αSyn clearance via the lysosomal pathway in αSyn transgenic mice [8]. These studies suggested that αSyn passive immunization ameliorates the degeneration in cellular and animal model of synucleinopathy. However, preclinical immunotherapies require long-term and repeated administration of antibodies to the animals. The large size of antibodies limits the ability to cross the blood-brain barrier. Combining gene and immunotherapy, we recently examined adeno-associated virus (AAV)-mediated gene transfer of αSyn antibodies in cellular and animal models [9]. We transduced the plasmids of intracellular single-chain intrabody [10] (NAC32, D10, or VH14) to HEK293 and SH-SY5Y cells. We demonstrated that the antibody targeting the 53–87 amino acid residues of human αSyn (NAC32) profoundly downregulated αSyn protein, but not αSyn mRNA levels in these cells. A similar response was also found in the Sprague-Dawley rats receiving intranigral administration of AAV-αSyn. AAV-NAC32 significantly reduced αSyn protein level in the nigra tissue. However, administration of AAV-αSyn nonselectively expressed and accumulated αSyn in dopaminergic and other cells in nigra. The expression of αSyn in nondopaminergic cells in nigra may hinder the behavioral phenotypes or pathology of PD. 

**Figure 1:**
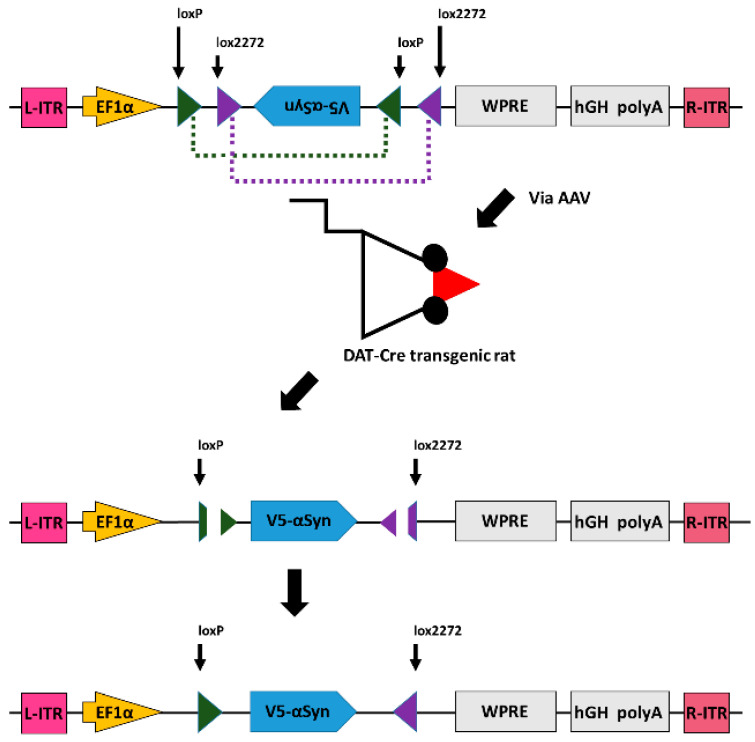
Expression of α-synuclein in dopaminergic neurons through Cre-DIO system. AAV containing the double floxed inverted open reading frame (DIO) of the α-synuclein (αSyn) construct, tagged with a V5 epitope at the N-terminal, was administered to the nigra of DAT-Cre rats, which constitutively express Cre recombinases driven by the promoter of dopamine transport (DAT) in dopaminergic neurons. DAT-specific Cre recombinase reverses the gene orientation of V5-αSyn in dopaminergic neurons via action on the lox2272 and loxP. Selective expression of V5-tagged αSyn can, thus, be established in nigral dopaminergic neurons of DAT-Cre transgenic rats. ITR, inverted terminal repeats; hGH poly A, human growth hormone polyadenylation signal; WPRE, woodchuck hepatitis virus post-transcriptional regulatory element.

In this study, we selective expressed αSyn in nigra DA neurons by local administration of AAV-DIO-αSyn to the nigra of DAT Cre rats [11,12], as seen in Figure 1. AAV containing the double floxed inverted open reading frame (DIO) of the αSyn construct was administered to the nigra of DAT-Cre rats, which constitutively express Cre recombinases driven by the promoter of dopamine transport (DAT) in dopaminergic neurons. DAT-specific Cre recombinase reverses the gene orientation of αSyn in dopaminergic neurons via acting on lox2272 and loxP. Selective expression of tagged αSyn can, thus, be established in nigral dopaminergic neurons of DAT-Cre transgenic rats. We next characterized the behavior response in these animals and expression of αSyn/TH in nigra dopaminergic neurons by immunohistochemistry. These animals developed bradykinesia and reduced TH immunoreactivity in SNcd, resembling αSyn-based dopaminergic degeneration in PD. Using this animal model, we demonstrated AAV-NAC32 selectively neutralized αSyn expression in nigra dopaminergic neurons and improved behavioral function and TH immunoreactivity in nigra.

## 2. Materials and Methods

### 2.1. Plasmid Construction

**Figure 2:**
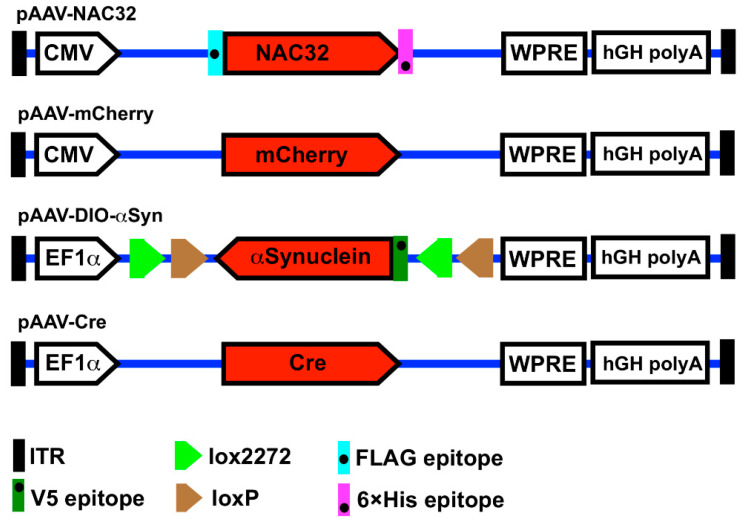
Illustration of adeno-associated virus vector plasmids. The pAAV-NAC32 plasmid encodes an αSyn-specific single-chain intrabody tagged with a FLAG epitope at the N-terminal and a 6×His epitope at the C-terminal. The pAAV-mCherry plasmid encodes a red fluorescence protein mCherry. The gene expression of these two constructs is driven by a promoter derived from cytomegalovirus. The pAAV-DIO-αSyn plasmid carries a double floxed inverted open reading frame (DIO) of the αSyn gene (tagged with a V5 epitope at the N-terminal) driven by the EF1-a promoter. The pAAV-Cre plasmid encodes a codon-improved Cre recombinase driven by the EF1-a promoter. The expression cassettes of these four constructs are flanked by the left- and right-inverted terminal repeat sequences (ITR) of serotype-2 adeno-associated virus. CMV, cytomegalovirus promoter; EF1a, elongation factor 1a promoter; lox2272, loxP, target sequences for the Cre recombinase; WPRE, woodchuck hepatitis B virus post-transcriptional regulatory element; hGH polyA, polyadenylation signal sequence of human growth hormone.

The genetic constructions of AAV vector plasmids pAAV-NAC32, pAAV-mCherry, pAAV-DIO-αSyn, and pAAV-Cre are illustrated in Figure 2. The DNA sequence (813 bp) encoding a single-chain intrabody (NAC32; tagged with FLAG and 6× Histidine at N-and C-terminals, respectively) against the nonamyloid component region of human αSyn was made by chemical synthesis (Genomics Ltd., New Taipei City, Taiwan) and then cloned into an AAV shuttle plasmid pAAV-MCS (Agilent Technologies, Santa Clara, CA, USA) at EcoRI and BamHI restriction enzyme sites. The DNA sequence (589 bp) of a post-transcriptional regulatory element derived from the woodchuck hepatitis B virus (WPRE; GeneBank accession # J04514) was synthesized and introduced into the 3’-end of NAC32 at BamHI and BglII sites, generating the vector plasmid pAAV-NAC32 [9]. The coding DNA sequence (711 bp) of the red fluorescence protein mCherry was synthesized and used to replace the NAC32 coding sequence on pAAV-NAC32 by cloning at EcoRI and BamHI sites resulting in the vector plasmid pAAV-mCherry. The coding DNA sequence of human αSyn tagged with a V5 epitope at the N-terminal was cloned in the inverted direction into an AAV shuttle plasmid at the location flanked by the lox2272+loxP sequence. The resulting vector plasmid pAAV-DIO-αSyn carries a double floxed inverted open reading frame and allows translation of N-terminal V5-tagged αSyn only in cells expressing Cre recombinase, which can reverse the orientation of a gene floxed by the lox2272+loxP sequence [13] (Figure 1). A Cre recombinase gene (1056 bp) with codon-optimization for the expression in mammalian cells [14] was cloned into an adeno-associated virus shuttle plasmid at BamHI and AscI sites, resulting in the vector plasmid pAAV-Cre. pAAV-DIO-αSyn and pAAV-Cre were kindly provided by Dr. Brandon K. Harvey (Optogenetic and Transgenic Technology Core, NIDA, NIH, Baltimore, MD, USA).

### 2.2. Virus Production and Titration

The recombinant AAV was generated by triple plasmid transfection [15]. On day 0, HEK293 cells (2 × 10^6^/dish; #240073, Agilent Technologies) were seeded in 20 culture dishes (15-cm diameter) and grown as monolayers in 20 mL of the growth medium [Dulbecco’s modified Eagle’s medium (DMEM) supplemented with 5% (*v/v*) heat-inactivated fetal calf bovine serum (FBS), penicillin (100 IU/mL), and streptomycin (100 mg/mL)] at 37 °C in a humidified incubator with 5% CO_2_. On day 4, the culture medium was replaced with 20 mL of fresh DMEM supplemented with 5% heat-inactivated FBS, and the subconfluent (70–80%) monolayer cells were co-transfected with (i) a vector plasmid pAAV-DIO-αSyn (9 μg), (ii) a capsid plasmid pRC1 (3.5 μg), and (iii) a helper-adenovirus plasmid pHelper (12.5 μg; Agilent Technologies) by the TransIT-X2 reagent (75 mL; #MIR6003, Mirus) in accordance with manufacturer’s instructions. The serotype-1 recombinant adeno-associated virus AAV-DIO-αSyn was generated by the HEK293 cells co-transfected with these three plasmids (Figure 2). For the production of other viral vectors (i.e., AAV-mCherry, AAV-NAC32, and AAV-Cre), the vector plasmid pAAV-DIO-αSyn was replaced with pAAV-mCherry, pAAV-NAC32, or pAAV-Cre in this co-transfection procedure (Figure 2). Those plasmids used in the AAV production were purified from *Escherichia coli* DH5a by the anion-exchange-based endotoxin-free plasmid purification kit (#12362, Qiagen, Hilden, Germany). At 16 h post-transfection, the culture medium was changed with 20 mL of fresh growth medium. On day 6 (48~58 h after transfection), cells were harvested by pipetting with the culture medium and then centrifuged at 2500× *g* at 4 °C for 10 min. Cell pellets were resuspended in the suspension buffer (1 mL for a pellet from a dish; 50 mM Tris-HCl PH8.0, 150 mM NaCl, 2 mM MgCl_2_), followed by freeze (−80 °C for 30 min) and thaw (37 °C for 15 min) for three rounds to burst open the cells. After centrifugation at 2500× *g* at 4 °C for 20 min, the supernatants of cell lysates were collected. In addition, the culture supernatants were mixed thoroughly with 40% PEG8000 (in 150 mM NaCl; # 1546605, Merck, St. Louis, MO, USA) to a final concentration of 8% and incubated at 4 °C for 2 h. The mixture was centrifuged at 2500× *g* at 4 °C for 20 min, and the pellet was dissolved in the suspension buffer at 1/80 of the starting volume. The supernatants of both cell lysates and culture media were combined and supplemented with Triton-X-100 (1%) and Benzonase (100 U/mL; #E1014, Sigma, St. Louis, MO USA). After incubation in a 37 °C water bath for 1 h, the mixture was centrifuged at 2500× *g* at 4 °C for 20 min, and the supernatant was filtered through a 0.2 mm filter cup. The AAV was purified using the HiTrap AVB affinity column (#GE28-4112-11, Cytiva, Uppsala, Sweden) equipped with a P1 peristaltic pump (#18111091, Cytiva, Upsala, Sweden) set at a flow rate of 2 mL/min. After equilibration with 20 mL of PBS, the column was loaded with the filtered supernatant (containing the AAV) and washed with 20 mL of PBS. The AAV was eluted from the column with 8 mL of glycine buffer (50 mM, PH2.7), and every 1 mL of elution fraction was collected in a microtube (containing 100 mL of 1 M Tris-HCl, PH8.0). The collected fractions with an O.D.280 value over 0.2 were pooled and concentrated in the Spin-X UF500 filter tube (100,000 MWCO; #431481, Corning, Leicestershire, UK) by centrifugation at 2500× *g* at 4 °C until the volume reduced by 90%. Subsequently, the filter tube was filled up with PBS and centrifuged again as described above. The concentrated viral suspension was filtered through a 0.2 mm filter disk, aliquoted (15 μL), and then stored at −80 °C until use.

Virus titers were determined by a quantitative real-time PCR assay (qPCR) performed on an ABI StepOnePlus system [15,16]. The purified virus sample (5 μL) was pre-treated with two units of DNase I (#M0303, New England BioLabs, Ipswich, MA, USA) in a final volume of 50 μL at 37 °C for 1 h, followed by incubation at 75 °C for 10 min to inactivate the DNase I activity. The primers that target the WPRE sequence on the vector plasmid to amplify a 384-bp fragment of the qPCR product were designed by the Primer-3 program and are listed as follows: 5’-TCATGCTATTGCTTCCCGTATGG-3’ (forward), 5’-GGATTGAGGGCCGAAGGGA-3’ (backward). Each qPCR reaction mixture (20 μL) contained 2 μL of DNase-pretreated virus sample, 10 mL of 2× SYBR green PCR master mix (#4367659, ABI), and 0.5 μM of each primer. Each reaction was triplicated in every qPCR assay. The PCR cycling program was set as the following: 95 °C for 10 min followed by 40 cycles for amplification (95 °C for 15 s, and 60 °C for 30 s), and a cycle for generating a melting curve (95 °C for 15 s, 60 °C for 1 min, and 95 °C for 15 s). A standard curve (plasmid copy numbers versus Ct values) using a ten-fold serial dilution (0.01–100 pg) of the vector plasmid was generated in every qPCR assay. Virus titers were calculated from this standard curve and expressed as viral genome copies per milliliter of the virus sample (VGC/mL).

### 2.3. Cell Culture and Immunocytochemistry

Hamster ovary CHO cells (2 × 10^5^ cells/well; #CCL-61, ATCC) were cultured on 12-mm glass coverslips in 24-well plates. Cells were incubated with Iscove’s Modified Dulbecco’s Medium (IMDM) supplemented with 10% (*v/v*) heat-inactivated fetal calf bovine serum (FBS), penicillin (100 IU/mL), and streptomycin (100 mg/mL) for 24 h at 37 °C and then were transduced with recombinant AAV-DIO-αSyn and/or AAV-Cre. At 48 h post-transduction, cells were washed with PBS, fixed with 4% formaldehyde for 10 min, and permeabilized with 0.3% Triton X-100 for 10 min. Cells were incubated with 4% FBS for 30 min to block nonspecific binding and then incubated with a mouse monoclonal antibody against V5 tag (1:250; #GTX42525, GeneTex, Irvine, CA, USA) for 1 h and with Alexa Fluor 488 goat anti-mouse IgG (1:250, ThermoFisher Scientific, Waltham, MA, USA) for 30 min at room temperature. The V5 immunoreactivity was examined using a fluorescence microscope.

### 2.4. Animals and Surgery

Adult male DAT-Cre rats of Long-Evan background (3-month-old, 336.0 ± 6.9 g) were kindly provided by the National Institute on Drug Abuse, NIH, and were bred at the National Health Research Institutes (Zhunan, Taiwan). Experimental procedures followed the guidelines of the “Principles of Laboratory Care” (National Institutes of Health publication no. 86–23, 1996) and were approved by the National Health Research Institutes (Taiwan) Animal Care and Use Committee (Protocols No.109057A, 105080A). Animals were housed in a 12-h dark (7 p.m. to 7 a.m.) and 12-hr light (7 a.m. to 7 p.m.) cycle. Animals were anesthetized and were placed in a stereotaxic frame. AAV-DIO-αSyn, AAV-mCherry, or AAV-NAC32 (2 μL of 1.0 × 10^12^ viral genomes/μL per site) was delivered bilaterally into to substantia nigra pars compacta dorsal tier (SNcd, AP−5.28 mm, ML: +/− 2.2 mm; DV:−7.9 mm to the bregma, based on Watson & Paxson’s rat brain atlas). The rate of infusion (1 μL/min) was adjusted by a microprocessor-controlled injector mounted to the stereotaxic frame (UMP4; World Precision Instruments, Sarasota, FL, USA). The needle remained in the brain for 2 min after the injection and then was slowly removed. After recovery from anesthesia, animals were housed in their home cages.

### 2.5. Locomotor Behavioral Measurement

Open-field locomotion was measured using an infrared activity monitor (Accuscan, Columbus, OH, USA). Animals were individually placed in the activity chambers (42 × 42 cm^2^) for 2 h (5 p.m. to 7 p.m.) for habituation. Locomotor parameters were recorded from 7 p.m. to 7 a.m. during the dark cycle. Food and water were available ad libitum. The following variables were measured: (i) Horizontal activity (HACTV, the total number of beam interruptions that occurred in the horizontal sensors), (ii) total distance traveled (TOTDIST, the distance, in centimeters, traveled by the animals), (iii) number of movements (MOVNO), (iv) movement time (MOVTIME), (v) rest time (RESTIME), and (vi) vertical activity (VACTV).

### 2.6. Immunohistochemistry

Animals were anesthetized and perfused transcardially with saline, followed by 4% paraformaldehyde (PFA) in phosphate buffer (PB; 0.1 M; PH 7.2). The brains were dissected, post-fixed in PFA for 18–20 h, and transferred to 20% sucrose in 0.1 M PB for at least 16 h. Serial sections of brains were cut at a 30 μm thickness on a cryostat (Leica, Model: CM 3050 S). Sections were rinsed with PB and were blocked with 4% BSA and 0.3% Triton X-100 in 0.1 M PB. Brain slices were then incubated with primary antibodies against tyrosine hydroxylase (monoclonal 1:200, Millipore, Billerica, MA, USA) or V5 tag (monoclonal 1:200, Invitrogen, Carlsbad, CA, USA) at 4 °C overnight. Sections were rinsed with 0.1 M PB and incubated in Alexa Fluor 488 secondary antibody solution (1:500, Invitrogen) and were mounted on slides and coverslipped. Confocal analysis was performed using a Nikon D-ECLIPSE 80 i microscope (Nikon Instruments, Inc., Tokyo, Japan) and EZ-C1 3.90 software (Nikon). Controls consisted of omission of the primary antibodies. TH and V5-αSyn-immunoreactivity in the SNcd were quantified by EZ-CI 3.90 software (Niko) and averaged in 3 adjacent brain slices between −4.80 to −5.04 mm posterior to bregma in all animals.

### 2.7. Statistical Analysis

All data were expressed as means ± SEM. Behavioral and biochemical data were analyzed using an unpaired *t*-test, or two-way ANOVA, and post-hoc Newman Keuls test (NK test). All analyses were calculated by Sigmaplot software v.12.5. Statistical significance was defined as *p* < 0.05.

## 3. Results

### 3.1. Expression of α-Synuclein by Cre-DIO in Cultured CHO Cells

**Figure 3:**
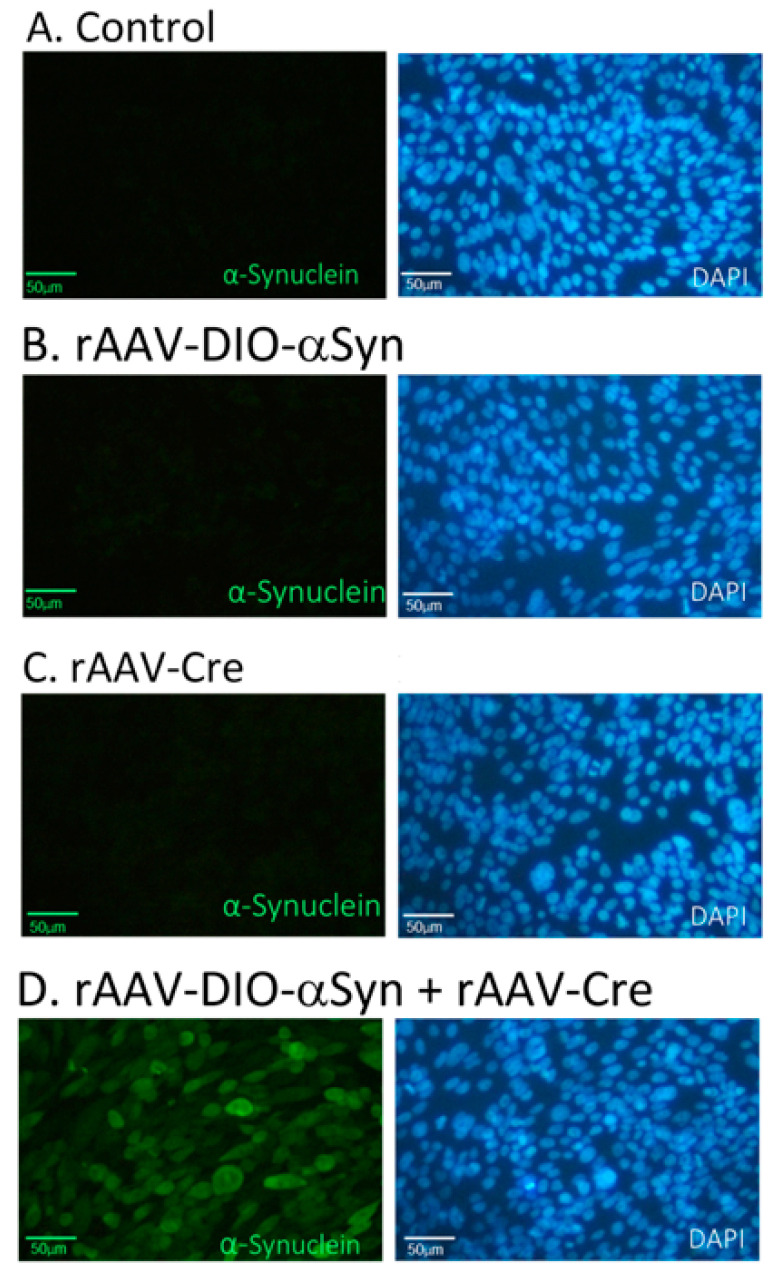
Expression of α-synuclein by Cre-DIO in cultured CHO cells. CHO cells were left untransduced (as a control; **A**) or transduced with the adeno-associated virus AAV-DIO-αSyn (**B**), AAV-Cre (**C**), or rAAV-DIO-αSyn+AAV-Cre (**D**). The expression of αSyn was identified by the presence of the V5 tag. V5-αSyn immunoreactivity (green) was found only in CHO cells co-transduced with AAV-DIO-αSyn and AAV-Cre. Cell nuclei were stained with DAPI (blue). Bar: 50 µm.

Cultured CHO cells were transduced with recombinant AAV-Cre and/or AAV-DIO-αSyn. Cells were fixed for immunocytochemistry 48 h later. The expression of αSyn was identified indirectly by the presence of the V5 tag. As seen in Figure 3, V5-tagged αSyn (V5-αSyn) immunoreactivity was found only in cells co-transduced with AAV-DIO-αSyn and AAV-Cre, indicating the successful expression of V5-αSyn after the interaction of Cre recombinase and the DIO gene construct.

### 3.2. Expression of αSyn in Nigral Dopaminergic Neurons In Vivo

**Figure 4:**
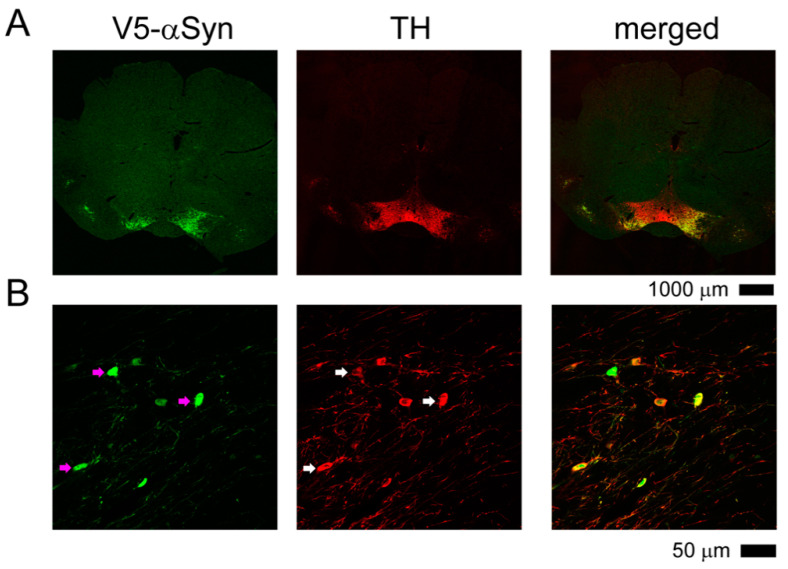
αSyn is expressed in nigral TH neurons of DAT-Cre rats receiving AAV-DIO-αSyn. DAT-Cre rats were stereotaxically injected with AAV-DIO-αSyn to SNcd. Brain tissues were collected for TH and V5-αSyn immunostaining. (**A**) At lower magnification, TH and V5-αSyn were present in the nigra region. (**B**) At higher magnification, almost all V5-αSyn (+) cells co-expressed TH immunoreactivity (arrows) in the SNcd.

Adult DAT-Cre rats were anesthetized and stereotaxically injected with AAV-DIO-αSyn into substantia nigra pars compacta dorsal tier (SNcd). Brain tissues (between −4.56 to −6.00 mm AP bregma) were collected for the expression of TH and V5-αSyn by immunohistochemistry at 12 weeks after viral injection. Both TH and V5-αSyn were found in the nigra region. All V5-αSyn (+) cells co-expressed TH immunoreactivity in the nigra (Figure 4). No V5-αSyn immunoreactivity was found in the control wild type (no Cre) rats receiving AAV-DIO-αSyn.

### 3.3. Overexpression of αSyn in Nigra Dopaminergic Neurons Induced Bradykinesia

**Figure 5:**
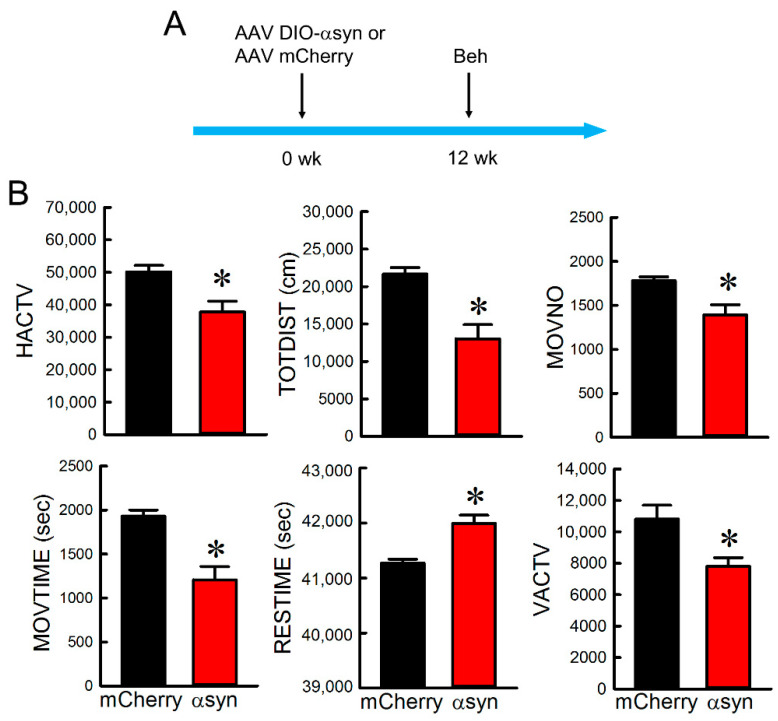
Administration AAV-DIO-αSyn to nigra induced bradykinesia in DAT-Cre rats. (**A**) Timeline of experiment. Animals receiving AAV-DIO-αSyn or AAV-mChery were placed in infrared locomotor activity chambers for 12 h (7 p.m. to 7 a.m.) during the dark cycle. (**B**) Intranigral administration of AAV-DIO-αSyn significantly reduced HACTV, TOTDIST, MOVNO, MOVTIME, and VACTV, while increased RESTIME. * Significant difference determined by *t*-test.

Adult male DAT-Cre rats receiving bilateral AAV-DIO-αSyn (*n* = 13) or AAV mCherry (*n* = 6) were used for the behavioral tests. Open-field locomotor activity was examined using an infrared activity monitor 12 weeks after the viral injection (see timeline in Figure 5A). Intranigral injection of AAV-DIO-αSyn significantly reduced HACTV (*p* = 0.0175), TOTDIST (*p* = 0.00394), MOVNO (*p* = 0.0226), MOVTIME (*p* = 0.00313), and VACTV (*p* = 0.00614), while it increased RESTIME (*p* = 0.00313; *t*-test, Figure 5B).

### 3.4. Intranigral Injection of AAV-NAC32 Normalized Locomotor Behavior

**Figure 6:**
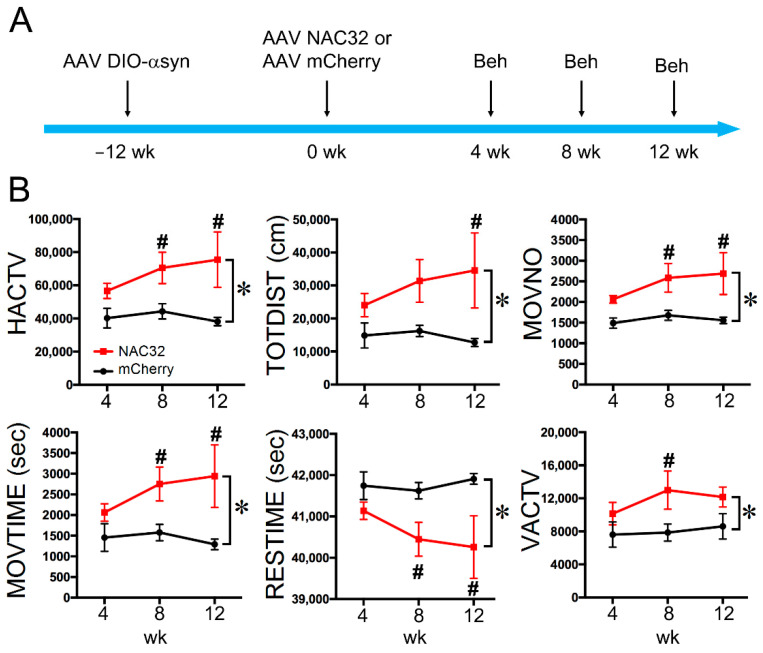
Intranigral administration of AAV-NAC32 normalized locomotor behavior in animals overexpressing αSyn. (**A**) Timeline of experiment. AAV-NAC32 or AAV-mCherry was injected into the SNcd of DAT-Cre rats at time 0. (**B**) Behavioral tests were conducted at 4, 8, and 12 weeks after the viral injection. Animals receiving AAV-NAC32 had a significant improvement in locomotor activity. The significant difference was determined by * two-way ANOVA and # post-hoc NK test.

At 12 weeks after AAV-DIO-αSyn injection, DAT-Cre animals received another dose of AAV-NAC32 (*n* = 4) or AAV-mCherry (*n* = 5) into the bilateral SNcd. Behavioral tests were conducted at 4, 8, and 12 weeks later (see timeline in Figure 6). Animals receiving AAV-NAC32 had a significant improvement in locomotor activity (Figure 6B, *p* < 0.05, two-way ANOVA+NK test). The detailed statistics are listed in Table 1.

### 3.5. AAV-NAC32 Increased TH While It Reduced αSyn Immunoreactivity in SNcd

**Figure 7:**
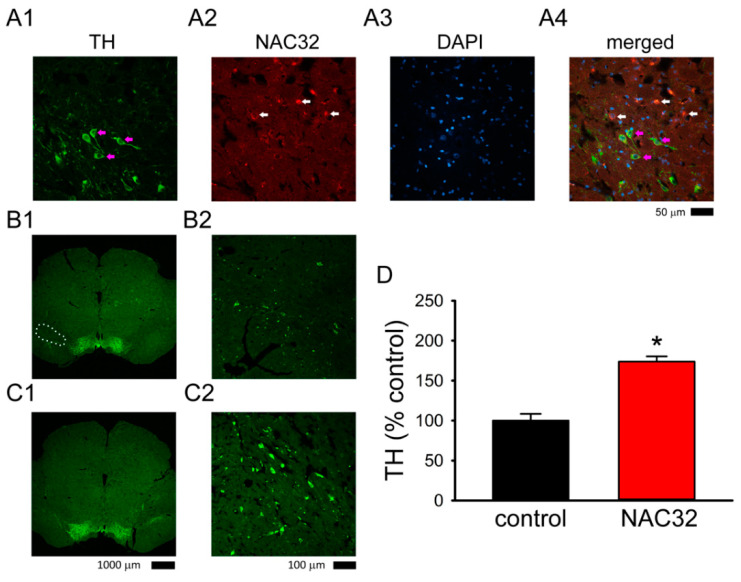
AAV-NAC32 reduced dopaminergic degeneration in nigra. (**A**) Representative photomicrographs of NAC32 and TH immunoreactivity (ir) in the SNcd in a DAT Cre rat receiving AAV-NAC32. NAC32-ir was found in the TH (purple arrows) and non-TH cells (white arrows) near the injection sites. (**A1**: TH, **A2**: NAC32, **A3**: DAPI, and **A4**: merged, **A1**–**A3**). (**B**) Animals receiving AAV-mCherry (**B1**, low magnification; **B2**, high magnification) had less TH immunoreactivity in the SNcd, comparing to (**C**) those receiving AAV-NAC32 (**C1**, low magnification; **C2**, high magnification). (**D**) TH immunoreactivity was averaged in the SNcd (dotted area in **B1**) of brain sections between −4.80 to −5.04 mm to the bregma. AAV-NAC32 significantly increased TH-ir in SNcd (* *p* < 0.001, *t*-test).

**Figure 8:**
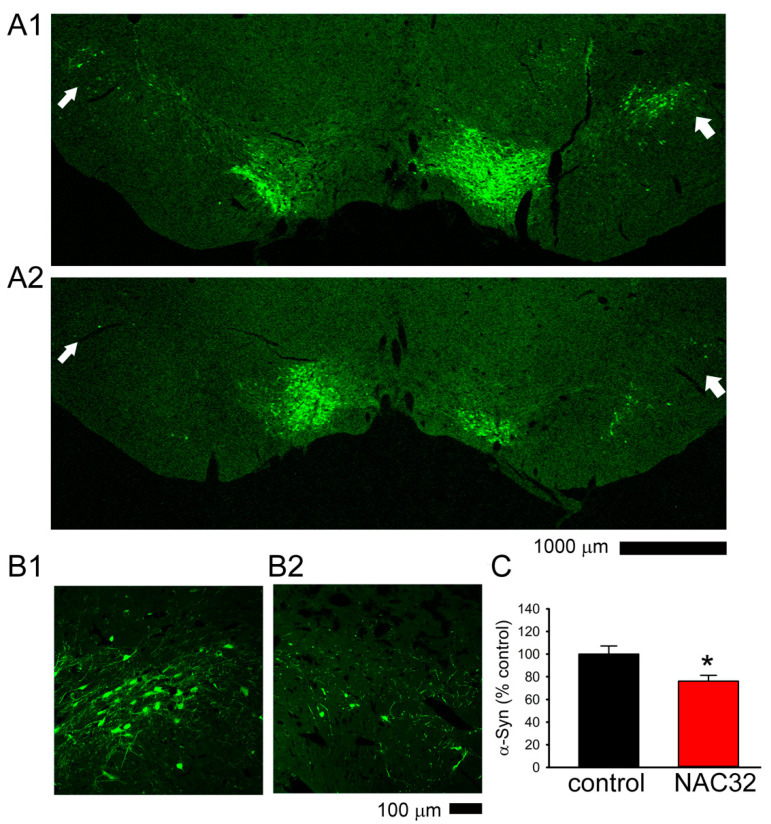
AAV-NAC32 reduced the expression of V5-αSyn in nigra. Representative photomicrographs of V5-αSyn-ir in the SNcd of animals receiving AAV-mCherry (**A1**, low magnification; **B1**, high magnification) or AAV-NAC32 (**A2**, low magnification; **B2**, high magnification). The expression of V5-αSyn in the SNcd (arrows, **A1** vs. **A2**; **B1** vs. **B2**) was reduced bilaterally after AAV-NAC32 injection. (**C**) V5-αSyn-ir was averaged in the SNcd (as seen in the dotted area in Figure 7B1). AAV-NAC32 significantly reduced V5-αSyn-ir in SNcd (* *p* = 0.018, *t*-test).

A total of 9 rats were used for TH and NAC32 immunostaining within 1 week after the last behavior test (see timeline, Figure 5A and Figure 6A). In animals receiving AAV-NAC32, the immunoreactivity (ir) of NAC32 was found in the TH and non-TH cells near the injection sites in SNcd (Figure 7A). No NAC32 activity was found in animals receiving AAV-mCherry. AAV-NAC32 increased TH-ir (Figure 7C: AAV-NAC32 vs. Figure 7B: AAV-mCherry) and reduced V5-αSyn expression in SNcd (Figure 8A2,B2): AAV-NAC32 vs. Figure 8A1,B1: AAV-mCherry). TH and V5-αSyn-ir in the SNcd were quantified and averaged from the brain sections between −4.80 to −5.04 mm posterior to bregma in all animals. A significant increase in TH-ir was found in animals receiving AAV-NAC than receiving AAV-mCherry (Figure 7D, *p* < 0.001, *t*-test). AAV-NAC32 significantly suppressed the expression of αSyn (Figure 8C, *p* = 0.018, *t*-test).

## 4. Discussion

In this study, selective expression of αSyn in the nigra dopaminergic neurons of DAT-Cre rats was carried out through local administration of AAV-DIO-αSyn. These animals developed PD-like phenotype, including bradykinesia and loss of dopaminergic neurons in nigra. Injection of AAV-NAC32 produced a selective antibody against the NAC in αSyn, normalized the behavior, and increased the survival of nigra dopaminergic cells. The main finding of this study is that AAV-NAC effectively antagonized αSyn-mediated dopaminergic degeneration in nigra.

Preferential degeneration of nigra dopaminergic neurons is a prominent character of PD and is frequently preceded by the accumulation of intracellular αSyn inclusions, such as Lewy bodies (LBs) and Lewy neurites (LNs) [17,18]. LBs and LNs are generated by the association of soluble αSyn into an insoluble aggregate core. Neurons with low levels of αSyn are spared from LB formation. Therefore, excessive intracellular production of αSyn is required for such aggregate formation and neuronal death [19,20], and has been used to model dopaminergic degeneration in PD [2,21]. Accumulation of αSyn can also occur in nondopaminergic neurons or peripheral tissue. For example, aggregation of αSyn in nigral nondopaminergic oligodendrocytes is associated with multiple system atrophy in patients [22,23,24]. The expression of αSyn in nondopaminergic cells in nigra may hinder the behavioral phenotypes or pathology of PD. In this study, αSyn was produced after co-transducing with AAV-Cre and AAV-DIO-αSyn in CHO cells. Using heterozygous rats expressing Cre-recombinase under dopamine transporter promotor, we selectively overexpressed αSyn in nigra dopaminergic neurons after local administration of AAV-DIO-αSyn. These animals developed bradykinesia and reduced TH immunoreactivity in SNcd, resembling αSyn-based dopaminergic degeneration in PD.

We previously demonstrated that transduction of NAC32, a single-chain antibody against the NAC of αSyn, downregulated αSyn protein in SH-SY5Y cells and adult Sprague Dawley rats nonselectively overexpressing αSyn in the nigra [9]. In this study, we characterized the behavior, and histological response of AAV-NAC32 in the animals selectively expressed αSyn in nigra DA neurons. AAV-NAC32 neutralized αSyn expression, antagonized locomotor deficits, and the loss of THir in DAT Cre rats expressing αSyn in nigra. These data support that AAV-NAC32 antibody selectively antagonized αSyn-mediated dopaminergic neurodegeneration.

Systemic passive αSyn antibody immunotherapy has been examined in animals and in patients. These antibodies effectively reduced αSyn aggregation in rodents [7]. In a phase 1 clinical trial, the αSyn monoclonal antibody PRX002 was found well-tolerated [25]. However, systemic passive antibody immunotherapy has some limitations. For example, long-term and repeated administration of antibodies is often required. The large size of antibodies limits the ability to cross the blood-brain barrier and reach the lesioned target. In contrast, in this study, the NAC32 antibody was produced and efficiently neutralized αSyn in the lesioned area in SNcd after a single dose of AAV-NAC32. We previously reported that administering a single dose rAAV vector carrying the gene for a methamphetamine monoclonal antibody (MethAb) resulted in long-term expression of the MethAb up to 30 weeks after injection in mice [15]. These data suggest that passive immunization through gene therapy may provide a long-term expression of antibodies. It will be of interest to further explore the duration of transgene expression in the host brain after AAV-NAC32 injection in future studies.

AAV was used to encode human αSyn fused with a V5 epitope at the N-terminal (V5-αSyn). V5 is a 14 amino acid epitope (GKPIPNPKKGLDST) identified from the V protein of simian virus 5 (SV5) and has been widely used as a peptide tag linked to a variety of recombinant proteins for the intracellular trafficking and isolation of tagged recombinant proteins [26,27]. The V5 tag can differentiate exogenous αSyn produced through viral infection and endogenous αSyn from the host, which does not contain the V5 tag. We reported that AAV-NAC32 mitigated V5-αSyn expression in the nigral dopaminergic neurons. As human and rat αSyn proteins have 95% identity and the majority (54–86 amino acid residues) of the NAC32 targeting epitope also exists on rat αSyn, it is possible that endogenous αSyn from rat may also be downregulated by AAV-NAC32, which warrants further investigation.

There are additional limitations to this study. Administration of AAV-NAC32 resulted in the production of neutralizing antibody and reduced the expression of αSyn in the lesioned target. These responses may be long lasting. In this study, the behavioral and neurodegenerative changes were monitored up to 12 weeks after AAV-NAC32 injection. A long-term follow-up is required to examine the side-effects of AAV-NAC32. To ensure local expression of NAC32, AAV-NAC32 was administered locally to the nigra. However, this approach still requires intracerebral surgery, which may limit its use in patients. Indeed, a full neutralizing antibody, as used here, would not readily cross an intact BBB. There are numerous studies to transiently open the BBB using various peptides or carrier molecules. However, these approaches having focused on antineoplastic drugs and how they would function with our antibody is not clear. An additional limitation is the appropriate target for neutralizing antibodies mature Lewy body inclusions or small oligomeric fibrils. These uncertainties may underlie the lack of clear positive findings in antibody clinical trials. A further ambiguity is the overall significance of the α synuclein target itself. Parkinson’s disease is associated with neuroinflammation, which is not well-targeted in α synuclein antibody clinical trials. An analogous problem exists in Alzheimer’s disease clinical trials with anti-amyloid antibodies.

## 5. Conclusions

In conclusion, we demonstrated that intracerebral administration of AAV-NAC32 expressed a selective αSyn antibody, through which the overexpressed αSyn protein in nigral dopaminergic neurons was neutralized, and dopaminergic function was preserved. Our data, thus, support that AAV-NAC32 can effectively antagonize αSyn-mediated dopaminergic degeneration in nigra in a rat PD model.

## Figures and Tables

**Table 1:** Significant improvement in locomotor behaviors after administration of AAV-NAC32 in rats overexpressing αSyn.

	* Two-Way ANOVA		Posthoc NK Test		
	*p*-Value	F Value	*p* at 4 wks	*p* at 8 wks	*p* at 12 wks
HACTV	<0.001	16.415	0.163	0.032	0.004
TOTDIST	0.002	12.18	0.259	0.059	0.009
MOVNO	<0.001	20.136	0.091	0.015	0.003
MOVTIME	0.001	14.088	0.254	0.038	0.005
RESTIME	0.001	14.09	0.254	0.038	0.005
VACTV	0.009	8.362	0.276	0.032	0.127

* Comparison was made in animals receiving AAV-NAC32 and AAV-mCherry from weeks 4 to 8. *p* and F values were determined by a two-way ANOVA+NK test. HACTV, horizontal activity; TOTDIST, total distance traveled; MOVNO, movement number; MOVTIME, movement time; RESTIME, rest time; VACTV, vertical activity.

## Abbreviations

αSyn	α-Synuclein
AAV	adeno associated virus
DA	dopaminergic
DAT	dopamine transporter
DAT-Cre rats	rats expressing Cre-recombinase under dopamine transporter promotor
DIO	double floxed inverted open reading frame
DMEM	Dulbecco’s modified Eagle’s medium
HACTV	horizontal activity
MOVNO	number of movements
MOVTIME	movements time
NAC	nonamyloidal component
NK test	Newman Keuls test
PD	Parkinson’s disease
RESTIME	rest time
SNcd	Substantia nigra pars compacta dorsal tier
TH	tyrosine hydroxylase
TOTDIST	total distance traveled
VACTV	vertical activity

## References

[1] Kruger R., Kuhn W., Muller T., Woitalla D., Graeber M., Kosel S., Przuntek H., Epplen J.T., Schols L., Riess O. (1998). Ala30Pro mutation in the gene encoding alpha-synuclein in Parkinson’s disease. Nat. Genet..

[2] Masliah E., Rockenstein E., Veinbergs I., Mallory M., Hashimoto M., Takeda A., Sagara Y., Sisk A., Mucke L. (2000). Dopaminergic loss and inclusion body formation in alpha-synuclein mice: Implications for neurodegenerative disorders. Science.

[3] Anderson E.N., Hirpa D., Zheng K.H., Banerjee R., Gunawardena S. (2019). The Non-amyloidal Component Region of alpha-Synuclein Is Important for alpha-Synuclein Transport Within Axons. Front. Cell Neurosci..

[4] Bisaglia M., Trolio A., Bellanda M., Bergantino E., Bubacco L., Mammi S. (2006). Structure and topology of the non-amyloid-beta component fragment of human alpha-synuclein bound to micelles: Implications for the aggregation process. Protein Sci..

[5] Oueslati A., Fournier M., Lashuel H.A. (2010). Role of post-translational modifications in modulating the structure, function and toxicity of alpha-synuclein: Implications for Parkinson’s disease pathogenesis and therapies. Prog. Brain Res..

[6] Allison J.R., Rivers R.C., Christodoulou J.C., Vendruscolo M., Dobson C.M. (2014). A relationship between the transient structure in the monomeric state and the aggregation propensities of alpha-synuclein and beta-synuclein. Biochemistry.

[7] Gupta V., Salim S., Hmila I., Vaikath N.N., Sudhakaran I.P., Ghanem S.S., Majbour N.K., Abdulla S.A., Emara M.M., Abdesselem H.B. (2020). Fibrillar form of alpha-synuclein-specific scFv antibody inhibits alpha-synuclein seeds induced aggregation and toxicity. Sci. Rep..

[8] Masliah E., Rockenstein E., Mante M., Crews L., Spencer B., Adame A., Patrick C., Trejo M., Ubhi K., Rohn T.T. (2011). Passive immunization reduces behavioral and neuropathological deficits in an alpha-synuclein transgenic model of Lewy body disease. PLoS ONE.

[9] Chen Y.H., Yu S.J., Wu K.J., Wang Y.S., Tsai H.M., Liao L.W., Chen S., Hsieh W., Chen H., Hsu S.C. (2020). Downregulation of alpha-Synuclein Protein Levels by an Intracellular Single-Chain Antibody. J. Parkinsons Dis..

[10] Lynch S.M., Zhou C., Messer A. (2008). An scFv intrabody against the nonamyloid component of alpha-synuclein reduces intracellular aggregation and toxicity. J. Mol. Biol..

[11] Schnutgen F., Doerflinger N., Calleja C., Wendling O., Chambon P., Ghyselinck N.B. (2003). A directional strategy for monitoring Cre-mediated recombination at the cellular level in the mouse. Nat. Biotechnol..

[12] Xu J., Zhu Y. (2018). A rapid in vitro method to flip back the double-floxed inverted open reading frame in a plasmid. BMC Biotechnol..

[13] Atasoy D., Aponte Y., Su H.H., Sternson S.M. (2008). A FLEX switch targets Channelrhodopsin-2 to multiple cell types for imaging and long-range circuit mapping. J. Neurosci. Off. J. Soc. Neurosci..

[14] Shimshek D.R., Kim J., Hübner M.R., Spergel D.J., Buchholz F., Casanova E., Stewart A.F., Seeburg P.H., Sprengel R. (2002). Codon-improved Cre recombinase (iCre) expression in the mouse. Genesis.

[15] Chen Y.H., Wu K.J., Wu K.L., Wu K.L., Tsai H.M., Chen M.L., Chen Y.W., Hsieh W., Lin C.M., Wang Y. (2017). Recombinant Adeno-Associated Virus-Mediated Expression of Methamphetamine Antibody Attenuates Methamphetamine-Induced Hyperactivity in Mice. Sci. Rep..

[16] Rohr U.P., Wulf M.A., Stahn S., Steidl U., Haas R., Kronenwett R. (2002). Fast and reliable titration of recombinant adeno-associated virus type-2 using quantitative real-time PCR. J. Virol. Methods.

[17] Schapira A.H. (1997). Pathogenesis of Parkinson’s disease. Bailliere’s Clin. Neurol..

[18] Spillantini M.G., Schmidt M.L., Lee V.M., Trojanowski J.Q., Jakes R., Goedert M. (1997). Alpha-synuclein in Lewy bodies. Nature.

[19] Volpicelli-Daley L.A., Luk K.C., Patel T.P., Tanik S.A., Riddle D.M., Stieber A., Meaney D.F., Trojanowski J.Q., Lee V.M. (2011). Exogenous α-synuclein fibrils induce Lewy body pathology leading to synaptic dysfunction and neuron death. Neuron.

[20] Taguchi K., Watanabe Y., Tsujimura A., Tanaka M. (2019). Expression of α-synuclein is regulated in a neuronal cell type-dependent manner. Anat. Sci. Int..

[21] Mori F., Nishie M., Kakita A., Yoshimoto M., Takahashi H., Wakabayashi K. (2006). Relationship among alpha-synuclein accumulation, dopamine synthesis, and neurodegeneration in Parkinson disease substantia nigra. J. Neuropathol. Exp. Neurol..

[22] Papp M.I., Kahn J.E., Lantos P.L. (1989). Glial cytoplasmic inclusions in the CNS of patients with multiple system atrophy (striatonigral degeneration, olivopontocerebellar atrophy and Shy-Drager syndrome). J. Neurol. Sci..

[23] Inoue M., Yagishita S., Ryo M., Hasegawa K., Amano N., Matsushita M. (1997). The distribution and dynamic density of oligodendroglial cytoplasmic inclusions (GCIs) in multiple system atrophy: A correlation between the density of GCIs and the degree of involvement of striatonigral and olivopontocerebellar systems. Acta Neuropathol..

[24] Gilman S., Wenning G.K., Low P.A., Brooks D.J., Mathias C.J., Trojanowski J.Q., Wood N.W., Colosimo C., Dürr A., Fowler C.J. (2008). Second consensus statement on the diagnosis of multiple system atrophy. Neurology.

[25] Jankovic J., Goodman I., Safirstein B., Marmon T.K., Schenk D.B., Koller M., Zago W., Ness D.K., Griffith S.G., Grundman M. (2018). Safety and Tolerability of Multiple Ascending Doses of PRX002/RG7935, an Anti-alpha-Synuclein Monoclonal Antibody, in Patients With Parkinson Disease: A Randomized Clinical Trial. JAMA Neurol.

[26] Southern J.A., Young D.F., Heaney F., Baumgärtner W.K., Randall R.E. (1991). Identification of an epitope on the P and V proteins of simian virus 5 that distinguishes between two isolates with different biological characteristics. J. Gen. Virol..

[27] Dunn C., O’Dowd A., Randall R.E. (1999). Fine mapping of the binding sites of monoclonal antibodies raised against the Pk tag. J. Immunol. Methods.

